# Involvement of toll-like receptor 2 and 4 in association between dyslipidemia and osteoclast differentiation in apolipoprotein E deficient rat periodontium

**DOI:** 10.1186/1476-511X-12-1

**Published:** 2013-01-08

**Authors:** Takaaki Tomofuji, Daisuke Ekuni, Tetsuji Azuma, Koichiro Irie, Yasumasa Endo, Kenta Kasuyama, Toshiki Yoneda, Manabu Morita

**Affiliations:** 1 Department of Preventive Dentistry, Okayama University Graduate School of Medicine, Dentistry and Pharmaceutical Sciences, 2-5-1 Shikata-cho, 700-8558, Okayama, Kita-ku, Japan

**Keywords:** Apolipoprotein E-deficient model, Alveolar bone, Toll-like receptor 2, Toll-like receptor 4, Osteoclast differentiation

## Abstract

**Background:**

Dyslipidemia increases circulating levels of oxidized low-density lipoprotein (OxLDL) and this may induce alveolar bone loss through toll-like receptor (TLR) 2 and 4. The purpose of this study was to investigate the effects of dyslipidemia on osteoclast differentiation associated with TLR2 and TLR4 in periodontal tissues using a rat dyslipidemia (apolipoprotein E deficient) model.

**Methods:**

Levels of plasma OxLDL, and the cholesterol and phospholipid profiles in plasma lipoproteins were compared between apolipoprotein E-deficient rats (16-week-old males) and wild-type (control) rats. In the periodontal tissue, we evaluated the changes in TLR2, TLR4, receptor activator of nuclear factor kappa B ligand (RANKL) and tartrate resistant acid phosphatase (TRAP) expression.

**Results:**

Apolipoprotein E-deficient rats showed higher plasma levels of OxLDL than control rats (p<0.05), with higher plasma levels of total cholesterol (p<0.05) and LDL-cholesterol (p<0.05) and lower plasma levels of high-density lipoprotein cholesterol (p<0.05). Their periodontal tissue also exhibited a higher ratio of RANKL-positive cells and a higher number of TRAP-positive osteoclasts than control rats (p<0.05). Furthermore, periodontal gene expression of TLR2, TLR4 and RANKL was higher in apolipoprotein E-deficient rats than in control rats (p<0.05).

**Conclusion:**

These findings underscore the important role for TLR2 and TLR4 in mediating the osteoclast differentiation on alveolar bone response to dyslipidemia.

## Background

Periodontal disease is a chronic inflammatory disease of the tooth-supporting structures. Studies have confirmed a positive association between periodontal disease and dyslipidemia. For instance, it has been reported that patients with dyslipidemia showed a significantly higher number of sextants with increased periodontal pockets than the control group [1]. It is also known that the ratio of high-density lipoprotein (HDL) to total cholesterol is associated with gingival index and percentage of bleeding on probing in patients with dyslipidemia [2]. Furthermore, it is shown that plasma level of lipid peroxidation was associated with the severity of periodontal disease in type 2 diabetes patients [3]. Although the cause of periodontal disease is oral bacterial pathogens (e.g., lipopolysaccharide [LPS]), it is conceivable that dyslipidemia contributes to the destructive aspects of the host response against bacterial pathogens that leads to periodontal disease, including alveolar bone loss.

Dyslipidemia increases the risk for overproduction of reactive oxygen species (ROS) in multiple organs [4], and excess ROS production impairs circulating oxidative/anti-oxidative balance that contributes to increased blood levels of oxidized low-density lipoprotein (OxLDL) [5]. Studies have demonstrated that OxLDL modulates toll-like receptor (TLR) expression and signaling [6,7]. TLR2 and TLR4 are critical receptors and signal transducers for oral bacterial LPS [8,9], and activate receptor activator of nuclear factor kappa B ligand (RANKL) expression [10], resulting in osteoclast differentiation in alveolar bone [11]. Therefore, it is possible that increased OxLDL following dyslipidemia advances periodontal disease through osteoclast differentiation via TLR signaling.

We previously found that diet-induced dyslipidemia is able to induce alveolar bone loss [12-14]. However, the role of TLR in dyslipidemia-induced alveolar bone loss has not been determined. In the present study, we hypothesized that the increased OxLDL in dyslipidemia may induce osteoclast differentiation on alveolar bone through TLR signaling. The apolipoprotein E (apoE)-deficient animal model is known to develop dyslipidemia [15-17]. Thus, the purpose of this study was to investigate the effects of dyslipidemia on osteoclast differentiation and TLR expression in periodontal tissue using an apoE-deficient rat model. To gain better insight into the mechanism of action, we analyzed histological changes and changes in TLR2, TLR4 and RANKL expression. In addition, expression of interleukin (IL)-1β in the periodontal tissue was evaluated as a parameter of periodontal inflammation activity.

## Materials and methods

### Animals

Control (wild-type) and dyslipidemic (apoE-deficient) rats (all Sprague–Dawley strain background; each group, n=6; age, 15 weeks) were obtained from Sigma Laboratory (St. Louis, MO). All experimental procedures were performed in compliance with guidelines approved by the Animal Research Control Committee of Okayama University Graduate School of Medicine, Dentistry and Pharmaceutical Sciences (OKU-2011004). Animals were maintained under standard conditions and given free access to food (MF; Oriental Yeast Co. Ltd., Osaka, Japan) and drinking water. The 7-day experiment was carried out at the same time for all groups. Collection of blood, sacrifice of rats and periodontal tissue isolation were performed as described previously [18].

### Histological analysis

For histological analysis, the maxillary molar regions (tooth and periodontal tissues) were resected *en bloc* from each rat and were immediately fixed in 4% paraformaldehyde in 0.1 mol/l phosphate buffer (pH 7.4) for 1 day. Tooth and periodontal tissues were decalcified with 10% tetrasodium-ethylenediaminetetraacetic acid aqueous solution (pH 7.4) for 8 weeks at 4°C. Paraffin-embedded bucco-lingual sections (4 μm) were stained with hematoxylin and eosin or other stains, as described below.

Immunostaining for lipid A, TLR2, TLR4 and RANKL were performed using a commercial kit (Histofine Simple Stain MAX PO; Nichirei Co., Tokyo, Japan). Monoclonal antibodies against TLR2 (Bioss Inc., Woburn, MA) and TLR4 (Santa Cruz Biotechnology, CA) and polyclonal antibody against RANKL (Santa Cruz Biotechnology) were diluted at 1/200, 1/300 and 1/100 in phosphate buffered saline, respectively. Color was developed by placing sections in a solution of 3-3’-diaminobenzidine tetrahydrochloride. To identify osteoclasts, tartrate-resistant acid phosphatase (TRAP) activity was also detected using the azo dye method [18]. Sections were counterstained with Mayer’s hematoxylin.

A single examiner (T. T.), blinded to the treatment assignment, performed histometric analyses using a microscope (Olympus Co., Tokyo, Japan). The distances between the cemento-enamel junction and the alveolar bone crest (an indicator of alveolar bone loss) were measured using a microgrid at a magnification of ×200 [12,18]. RANKL-positive osteoblasts, total osteoblasts and TRAP-positive osteoclasts on the surface of alveolar bone were counted using a light microscope at ×400 magnification and are reported in terms of number/millimeters [13,14]. We evaluated intra-examiner reproducibility by double-scoring 10 randomly selected sections at two-week intervals. Agreement for RANKL-positive cells and TRAP-positive osteoclasts was more than 90%.

### Gene expression analyses

Total RNA was extracted from periodontal tissue samples using a commercial reagent (Invitrogen). The purity of mRNA was determined by 260/280 nm absorbance ratio, and only samples with a ratio greater than 1.8 were used. Samples (2 μg) of total RNA from each group, which was reverse transcribed by AMV Reverse Transcriptase (TAKARA, Kyoto, Japan) at 42°C for 30 min, were used to perform first-strand cDNA synthesis using a commercial kit (Roche, Tokyo, Japan). cDNA prepared as described above was diluted 10-fold with yeast RNA (10 μg/mL). Primer sequences for the genes encoding rat TLR2, TLR4, RANKL, IL-1β and β-actin are shown in Table 1. Cycling conditions using TOYOBO SYBR Green PCR Master Mix (TOYOBO, Osaka, Japan) in a LightCycler™ (Roche Applied Science, Mannheim, Germany) system were as follows: 95°C for 30 s, 59°C for 30 s and 72°C for 30 s for 45 cycles. mRNA levels were expressed in terms of relative copy number ratio for TLR2, TLR4, RANKL or IL-1β against β-actin for each sample. Gene expression in the dyslipidemia group was calculated in terms of relative copy number ratio for each mRNA against the control group for each sample.

**Table 1:** Primer sequences for IL-1β, RANKL, TLR2, TLR4 and β-actin

	**Sense (5’-3’)**	**Antisense (3’-5’)**	**Length (bp)**	**Accession No.**
IL-1β	CACCTCTCAAGCAGAGCACAGA	CTGAAGTGGTACCTTGGGCA	81	NM031512
RANKL	GCTCACCTCACCATCAATGCT	ATTTCAGTCAGACAGGAGAACCATGG	70	NM057149
TLR2	TCTGAGTTCCGTGACATAGG	AGATGTAACGCAACAGATTC	169	NM198769
TLR4	GTGAGCATTGATGATGAGTTCAG	CATCTAATGATTGATAAGGATT	170	NM019178
β-actin	TGTTGCCCTAGACTTCGAGCA	GGACCCAGGAAGGAAGGCT	155	NM007393

### Analysis of blood samples

Blood samples were collected from the heart at age 8 weeks. Blood was allowed to clot at room temperature, and plasma was separated by centrifugation at 1,500 ×*g* for 15 min. Levels of plasma OxLDL were measured using an ELISA kit (Cusabio Biotech Co., Ltd., Wuhan, China) [19]. The cholesterol and phospholipid profiles in plasma lipoproteins were analyzed using a dual detection high-performance liquid chromatography system at Skylight Biotech (Akita, Japan) [20]. We quantified individual subfractions using best curve fitting analysis, assuming that the particle sizes for all subfractions followed a Gaussian distribution. Particle sizes for individual subfractions were determined as 30–80 nm (very low-density lipoprotein [VLDL]), 16–30 nm (LDL) and 8–16 nm (HDL).

### Statistical analysis

All data are expressed as means ± S.D. Comparisons between the dyslipidemia and control groups were performed by independent *t*-test using a statistical software package (SPSS 17.0 J for Windows, IBM, Tokyo, Japan). A p value of < 0.05 was accepted as statistically significant. Sample size was calculated using the nQuery Advisor (Statistical Solutions, Saugus, MA), based on the results of our previous studies [13,14]. A sample size of 6 per group was required for detection of significant differences (80% power, two-sided, 5% significance level).

## Results

No significant differences were observed between the control and dyslipidemia groups with regard to food consumption, body weight or growth pattern during the 7-day period.

**Figure 1:**
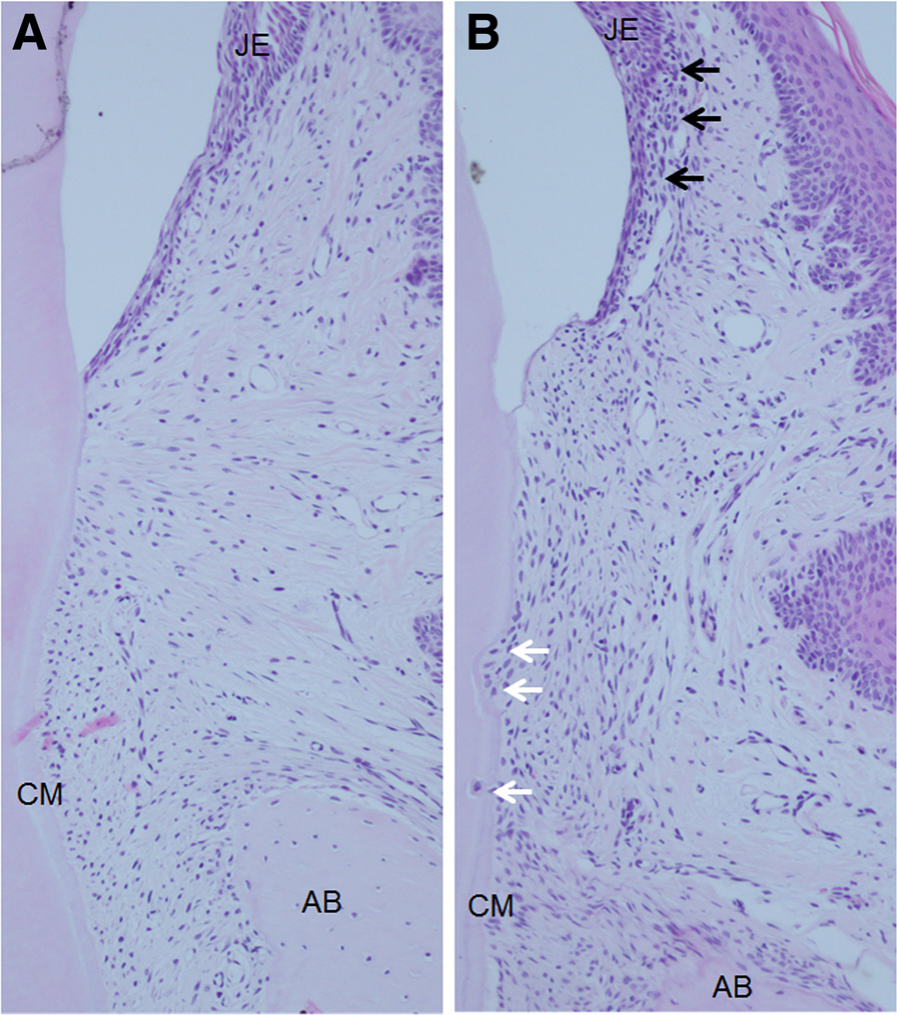
**Hematoxylin and eosin staining of rat periodontal tissue at 7 days.** No pathological changes were observed in the periodontium in any of the samples in the control group (**A**). The dyslipidemia group showed extension of blood vessels, root resorption (white arrows) and the increased number of inflammatory cells subjacent to the junctional epithelium (JE) (black arrows) (**B**). AB, alveolar bone and CM, cementum. Original magnification × 10.

**Figure 2:**
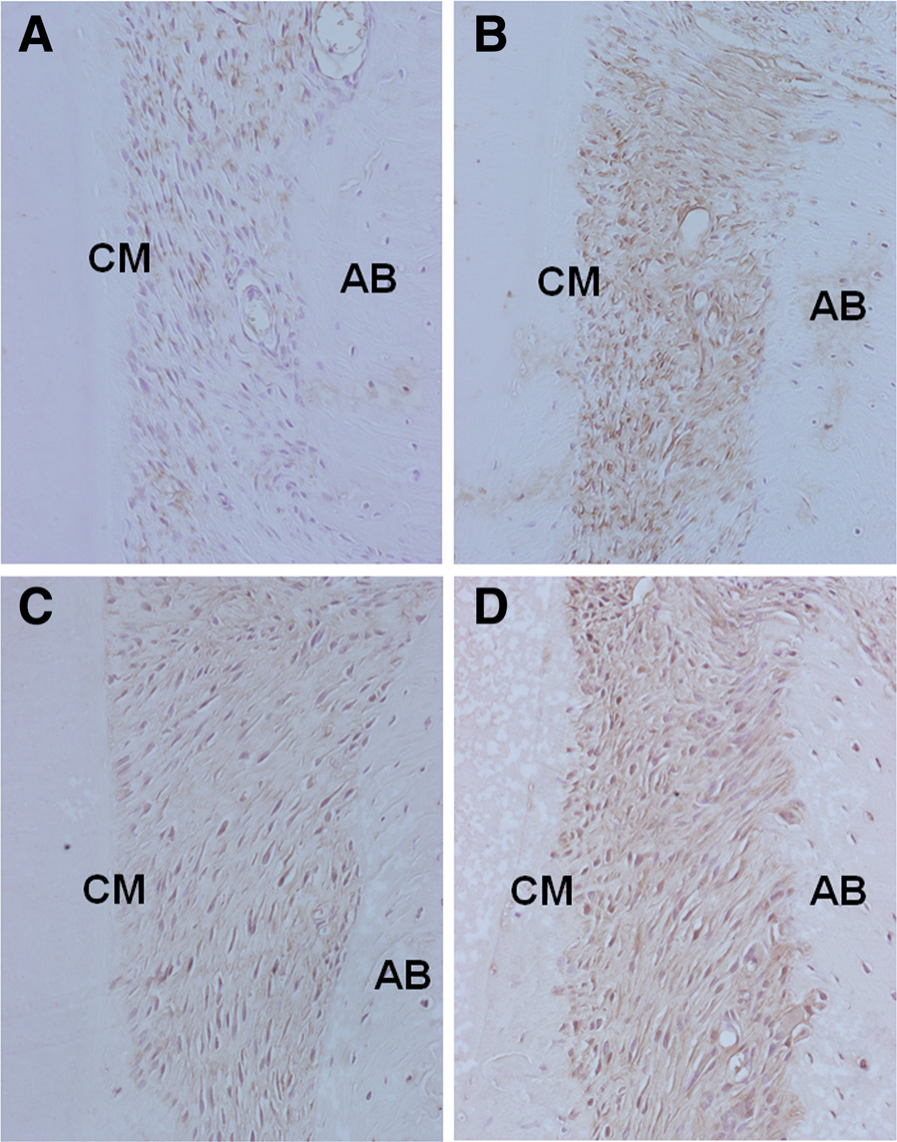
**Immunohistochemical staining for TLR-2 (A and B) and TLR-4 (C and D) in the periodontal tissue.** The dyslipidemia group (**B** and **D**) showed higher expressions of TLR2 and TLR4 compared to the control group (**A** and **C**), respectively. Original magnification × 20.

**Figure 3:**
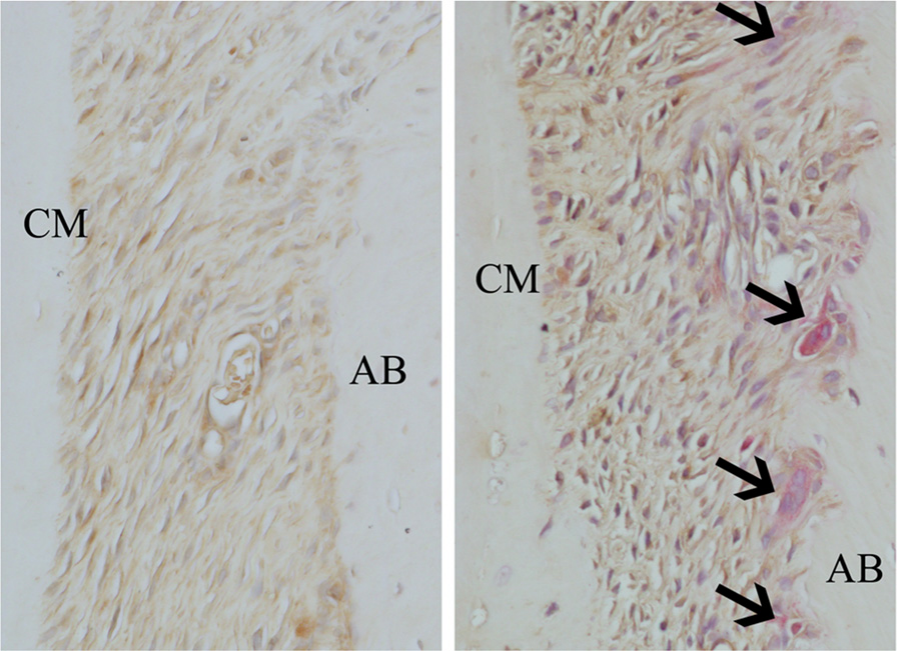
**Immunohistochemical staining for RANKL and TRAP in the periodontal tissue.** The dyslipidemia group (**B**) showed higher expressions of RANKL and TRAP (arrows) compared to the control group (**A**). Original magnification × 40.

In the dyslipidemia group, periodontal tissues showed extension of blood vessels, root resorption and increased numbers of inflammatory cells; however, the control group showed no pathological changes at 7 days (Figure 1). The numbers of polymorphonuclear leukocytes, RANKL-positive osteoblasts and TRAP-positive osteoclasts in the dyslipidemia group were also higher than those in the control group (p< 0.05) (Table 2). Furthermore, the periodontium in the dyslipidemia group showed high expression of TLR2, TLR4, RANKL, and TRAP, as compared to the control group (Figures 2 and 3). In addition, although the dyslipidemia group showed greater distances between the cemento-enamel junction and alveolar bone crest than the control group, this difference was not statistically significant.

**Table 2:** Histopathological evaluation in periodontal tissues (mean ± SD)

	**Control (N= 6)**	**Dyslipidemia (N= 6)**
Polymorphonuclear leukocytes (numbers/0.05 mm x 0.1 mm)	0.7 ± 0.5	2.1 ± 0.5^a^
Distance between the cemento-enamel junction and the alveolar bone crest (μm)	571 ± 100	618 ± 42
Ratio of RANKL-positive cells	0.18 ± 0.05	0.41 ± 0.07^a^
TRAP-positive osteoclasts (numbers/mm)	2.3 ± 2.2	7.9 ± 2.7^a^

^a^ Significantly different from the Control group, p < 0.05 (*t*-test).

**Figure 4:**
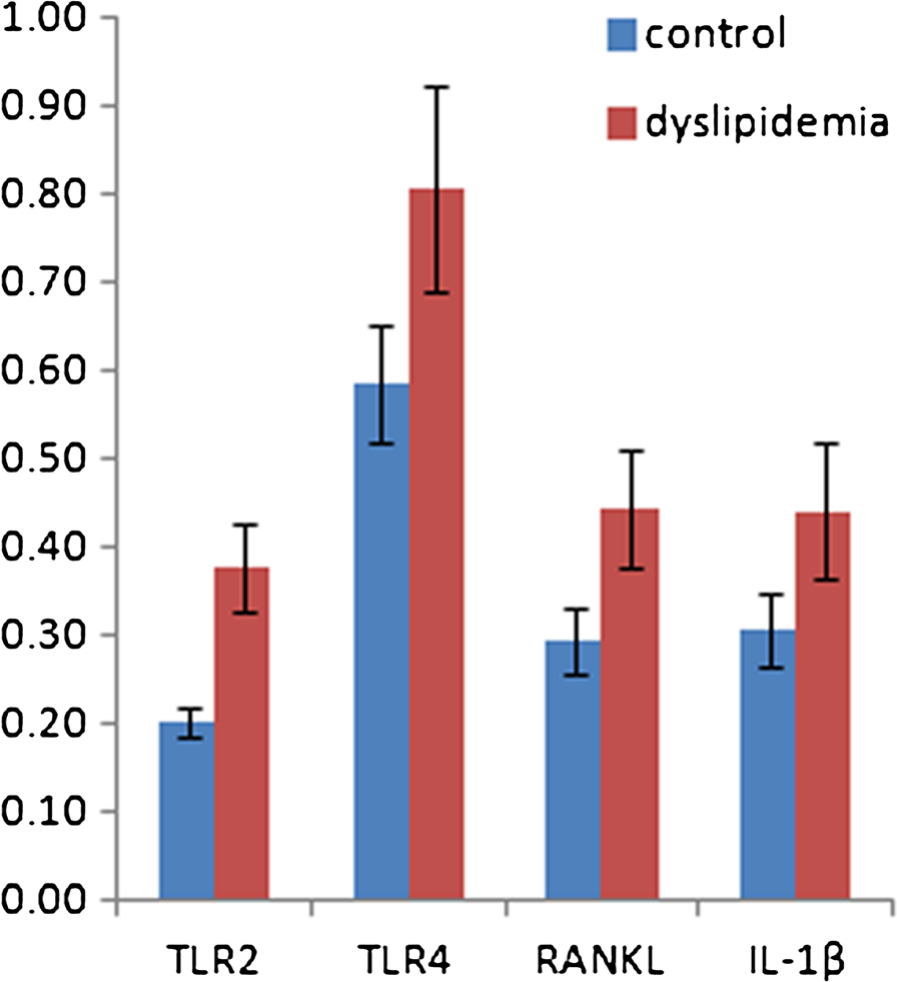
**Fold changes of gene expression on rat periodontal tissue (mean ± SD).** The mRNA levels were calculated in terms of the relative copy number ratio of each mRNA to β-actin for each sample (n=6 per group). All values in the dyslipidemia group were significantly higher than those in the control group (p < 0.05).

Gene expression of TLR2, TLR4 and RANKL was 1.90, 1.37, and 1.52 times higher in periodontal tissues obtained from the dyslipidemia group than in those obtained from the control group, respectively (p < 0.05) (Figure 4). Gene expression of IL-1β in periodontal tissues was also higher in the dyslipidemia group than that in the control group (p < 0.05).

At 7 days, plasma levels of OxLDL were 2.31 times higher in the dyslipidemia group than in the control group (p < 0.05) (Table 3). Plasma levels of total cholesterol, VLDL cholesterol and LDL cholesterol were also higher in the dyslipidemia group than in the control group (p < 0.05). On the other hand, plasma HDL cholesterol levels were lower in the dyslipidemia group than in the control group (p < 0.05).

**Table 3:** Plasma levels of lipoproteins and oxLDL in rats (mean ± SD)

	**Control (N= 6)**	**Dyslipidemia (N= 6)**
Total cholesterol (mg/dL)	40.8 ± 6.6	130.8 ± 25.0^a^
VLDL cholesterol (mg/dL)	7.1 ± 1.2	54.2 ± 11.6^a^
LDL cholesterol (mg/dL)	13.6 ± 1.9	51.4 ± 11.6^a^
HDL cholesterol (mg/dL)	31.5 ± 4.9	10.2 ± 1.3^a^
OxLDL (ng/mL)	13.7 ± 3.0	31.7 ± 6.3^a^

^a^ Significantly different from the control group, p < 0.05 (*t*-test).

## Discussion

The apoE-deficient rats showed higher plasma levels of total cholesterol, LDL cholesterol and VLDL cholesterol, and lower plasma levels of HDL cholesterol, as compared to control rats. Thus, the apoE-deficient rats exhibited dyslipidemia in this study. In addition, the periodontal tissues in the apoE-deficient rats exhibited higher expression levels of TLR2, TLR4, RANKL and TRAP than those in the control rats. TLR activates RANKL expression [10], resulting in osteoclast differentiation [11]. It is possible that dyslipidemic conditions are associated with TLR-induced osteoclast differentiation in periodontal tissues.

Previous studies have demonstrated that OxLDL has direct effects on TLR2 and TLR4 expression [6,7]. In this study, dyslipidemic conditions induced higher plasma levels of OxLDL than normal conditions. Increased plasma levels of OxLDL following dyslipidemic conditions may induce increased expression of TLR2 and TLR4. TLR 2 and 4 are critical receptors for oral bacterial LPS [8,9]. Dyslipidemic conditions would alter the interaction between bacterial pathogens and the cellular membrane, by increasing plasma levels of OxLDL.

In this study, dyslipidemic conditions were associated with higher plasma levels of total cholesterol, LDL cholesterol and VLDL cholesterol, and lower plasma levels of HDL cholesterol when compared with normal conditions. In clinical studies, it has been shown associations between blood lipid parameters and periodontal condition [21-23]. Therefore, it is also possible that impaired plasma lipids had direct effects on TLR2 and TLR4 expression in the current model. However, we previously found that changes in osteoclast differentiation under hypercholesterolemic conditions did not depend on blood lipids [13,24]. This suggests that impaired lipids do not play a crucial role in TLR2 and TLR4 expression in the periodontal tissue.

It has been reported that activation of TLR2 and TLR4 promotes expression of inflammatory cytokines [25,26]. In the present study, dyslipidemic conditions induced gene expression of IL-1β, an inflammatory cytokine. Inflammatory cytokines promote osteoclast differentiation both directly and indirectly [27]. These observations indicate that the increased osteoclast differentiation under dyslipidemic conditions is partly caused by induction of inflammatory cytokines. Furthermore, overproduction of inflammatory cytokines can stimulate inflammatory responses [28], and this would advance periodontal inflammation. In fact, inflammatory histological changes, such as increased number of polymorphonuclear leukocytes and root resorption, were also observed in the current dyslipidemia model.

In our previous study [14], hypercholesterolemic rats (age, 20 weeks) fed a high-cholesterol diet for 8 weeks showed increased numbers of RANKL-positive cells on the alveolar bone surface. In that study, the ratio of RANKL-positive cells to total cells (mean ± SD) was 0.15 ± 0.04 in the control group and 0.32 ± 0.10 in the hypercholesteromic group [14]. In the present study using an apoE-deficient model (age, 16 weeks), the ratio of RANKL-positive cells to total cells (mean ± SD) was 0.18 ± 0.05 in the control group and 0.41 ± 0.07 in the dyslipidemic group. These results are in agreement with previous findings that impaired lipid metabolism induces RANKL expression.

Studies have shown positive association between dyslipidemia and periodontal disease [21,29,30]. In our previous animal study, feeding with a high-cholesterol diet increased serum lipid peroxidation and the number of TRAP-positive osteoclasts, with an increase in RANKL expression [14]. In this study, we demonstrated that activation of TLR2 and TLR4 is involved in osteoclast differentiation on the surface of alveolar bone under dyslipidemic conditions. Therefore, suppression of TLR expression may be an effective method for preventing osteoclast differentiation under dyslipidemic conditions. However, further studies are necessary.

Our study has several limitations. As the bacterial flora in the gingival sulcus of rats differs from that in humans, we did not investigate how dyslipidemic conditions directly affect bacterial flora. More studies are thus necessary in order to clarify this issue. In addition, although osteoclast differentiation increased under dyslipidemic conditions, the linear distance between the cemento-enamel junction and alveolar bone crest did not change in our model. As the experimental period was only 7 days, long-term analysis is necessary.

In conclusion, dyslipidemia induces osteoclast differentiation on the alveolar bone surface by activation of TLR2 and TLR4 in the rat apolipoprotein E knockout model.

## Abbreviations

Apo E: Apolipoprotein E;HDL: High-density Lipoprotein;LPS: Lipopolysaccharide;IL: Interleukin;LDL: Low-density Lipoprotein;OxLDL: Oxidized Low-density Lipoprotein;RANKL: Receptor Activator of Nuclear Factor Kappa B Ligand;ROS: Reactive Oxygen Species;TRAP: Tartrate Resistant Acid Phosphatase;TLR: Toll-like Receptor;VLDL: Very low-density Lipoprotein

## Competing interests

The authors declare that they have no competing interests.

## Authors’ contributions

Conceived and designed the experiments: TT and MM. Performed the experiments: TT, KI, KK, and TY. Analyzed the data: DE and YE. Wrote the paper: TT and MM. All authors read and approved the final manuscript.
